# The Experience of Locomotor Training From the Perspectives of Therapists and Parents of Children With Cerebral Palsy

**DOI:** 10.3389/fresc.2021.740426

**Published:** 2021-12-02

**Authors:** Dayna Pool, Catherine Elliott, Claire Willis, Ashleigh Thornton

**Affiliations:** ^1^School of Allied Health, Curtin University, Perth, WA, Australia; ^2^The Healthy Strides Foundation, Perth, WA, Australia; ^3^Telethon Kids Institute, Perth, WA, Australia; ^4^Perth Children's Hospital, Perth, WA, Australia; ^5^School of of Allied Health, Human Services and Sport, La Trobe University, Melbourne, VIC, Australia; ^6^UWA Medical School, The University of Western Australia, Perth, WA, Australia

**Keywords:** qualitative, locomotor training, cerebral palsy, physical activity, sedentary

## Abstract

**Objective:** The objective of this study was to explore the experiences of intensive locomotor training from the perspective of therapists and parents of children with cerebral palsy.

**Design:** A qualitative study using semi-structured interviews was employed to capture perspectives following an intensive locomotor training intervention. Data were analyzed thematically, systematically coding and interpreted by grouping information into themes and sub-theme categories.

**Participants:** Five therapists and seven parents of children with high daily physical assistance and equipment needs participated in the study.

**Setting:** A pediatric tertiary hospital.

**Results:** Experiences of locomotor training were described with relation to the suitability of locomotor training with sub-themes of intervention length and time, engagement within sessions, the importance of support, and the utility of locomotor training beyond a research context. Motivation for participating in locomotor training was described in relation to the enjoyment of movement and for increasing activity level. The barriers and facilitators who participated in locomotor training provided environmental and personal factor subthemes. Finally, the outcomes from the intervention were related to improvements in physical health, sleep, affect and emotion, and ambulation in daily activities.

**Conclusion:** The experience of intensive locomotor training from the perspectives of parents of children who have high physical assistance and equipment needs and the therapists providing the intervention was described. Future studies should consider outcome measures beyond motor capacity to quantify the perceived outcomes of interventions that are meaningful to families.

## Introduction

Cerebral palsy (CP) is a complex life-long neurological condition primarily affecting movement and posture. It is the most common cause of physical disability in childhood (1). For children and youth who are dependent on physical assistance and equipment for mobility throughout the day, there are limited evidence-based interventions available that aim to increase physical activity and improve gross motor function (2). This is despite secondary complications of inactivity and physical deterioration that most notably occurs between the ages of 7 to 9 years (3). These children and youth are described under the Gross Motor Function Measure Classification System (GMFCS) as being levels III (ambulators with assistance), IV (non-ambulant but able to sit unassisted), and V (non-ambulant and unable to sit) (4). For this group of children and youth, the combination of limited evidence-based interventions and physical deterioration significantly impacts quality of life and overall health and well-being (5).

Locomotor training is an activity-based approach that aims to support the development of stepping skills in individuals with significant gross motor limitations (6, 7). Locomotor training is usually delivered in two parts, first through partial body weight supported treadmill training (PBWSTT) to enable individuals to be supported in the development of stepping whilst managing less of their own body weight (8). A harness is used to support the safe attainment of a more upright position with reported benefits in walking speed, endurance, and potential efficacy in children with more severe physical limitations (9–11). Second, overground walking practice is also incorporated into locomotor training providing task specific whole-task practice (8). For children classified within GMFCS levels III, IV, and V, PBWSTT and overground walking practice have strong recommendations for the outcomes of improving walking distance, providing the experience of walking for well-being, and inclusion and for improving transfer abilities (12). With the growth of new technologies, locomotor training has also expanded to include robotic assistive gait training (RAGT). The use of RAGT is usually adopted to increase engagement and provide a higher dosage of training with less therapist involvement (13). Both PBWSTT and RAGT present as viable options to facilitate engagement in physical activity particularly for children with CP who have more physical limitations. Typically, engaging in locomotor training for individuals with neurological conditions requires a higher dosage of treatment where attendance occurs over several sessions a week for a number of weeks (14). This is mainly based on current recommendations for exercise and physical activity prescription for children with CP (15, 16).

Between June 2015 and January 2017, a clinical trial co-designed by consumers known as iStride was conducted in a pediatric tertiary hospital in Perth, Australia. The aim of the trial was to determine if the addition of RAGT (utilizing the RT600, Restorative Therapies, Baltimore, MD, USA) to PBWSTT improved motor outcomes compared to PBWSTT alone in a randomized controlled trial. This trial recruited 40 participants aged between 5 and 12 years and classified them as functioning at GMFCS levels III, IV, and V. The intervention involved a high dosage of treatment with participants attending three 1 h sessions a week for 6 weeks (17). As such, evaluating the experience of this high dosed intervention from the perspectives of parents, children, and therapists involved in the intervention was considered vital.

Although service providers endeavor to deliver holistic, strength-based interventions (18), there is a paucity of qualitative reports on the outcomes of intensive therapy models and, in particular, locomotor training in children with CP functioning within GMFCS levels III, IV, and V. Qualitative approaches can provide evidence related to the experience of an intervention (16), insight into the value that different stakeholders attach to different intervention outcomes, and uncover considerations relevant to implementation and practice (19). Understanding the experiences of locomotor training through a strengths-based lens, which is considered essential in childhood disability (18), ensures that the evidence-base represents outcomes that are meaningful to therapists, children, and their families.

Therefore, this qualitative study aimed to explore the experience of intensive locomotor training from the perspectives of parents of children and youth with CP (GMFCS levels III-IV-V) and therapists. Specifically, we aimed the following:

1. Describe the outcomes of locomotor training from the perspective of parents, children, and therapists providing the intervention and;
2. Inform future best practice care and research for children functioning with GMFCS levels III, IV, and V.

## Methods

### Design

A qualitative description approach was employed to evaluate the iStride randomized controlled trial (https://www.anzctr.org.au/ Trial number: ACTRN12615001149550). Contrasted with other qualitative approaches that interpret the meaning or develop theory, the goal of qualitative description was to provide a rich and clear description of an experience or process to inform and improve healthcare (20, 21). Human ethics approval was obtained from the Human Research Ethics Committees of Perth Children's Hospital and Curtin University, Perth Australia. Written informed consent and assent for participant and publication was obtained from children and their parents.

### Participants

**Figure 1:**
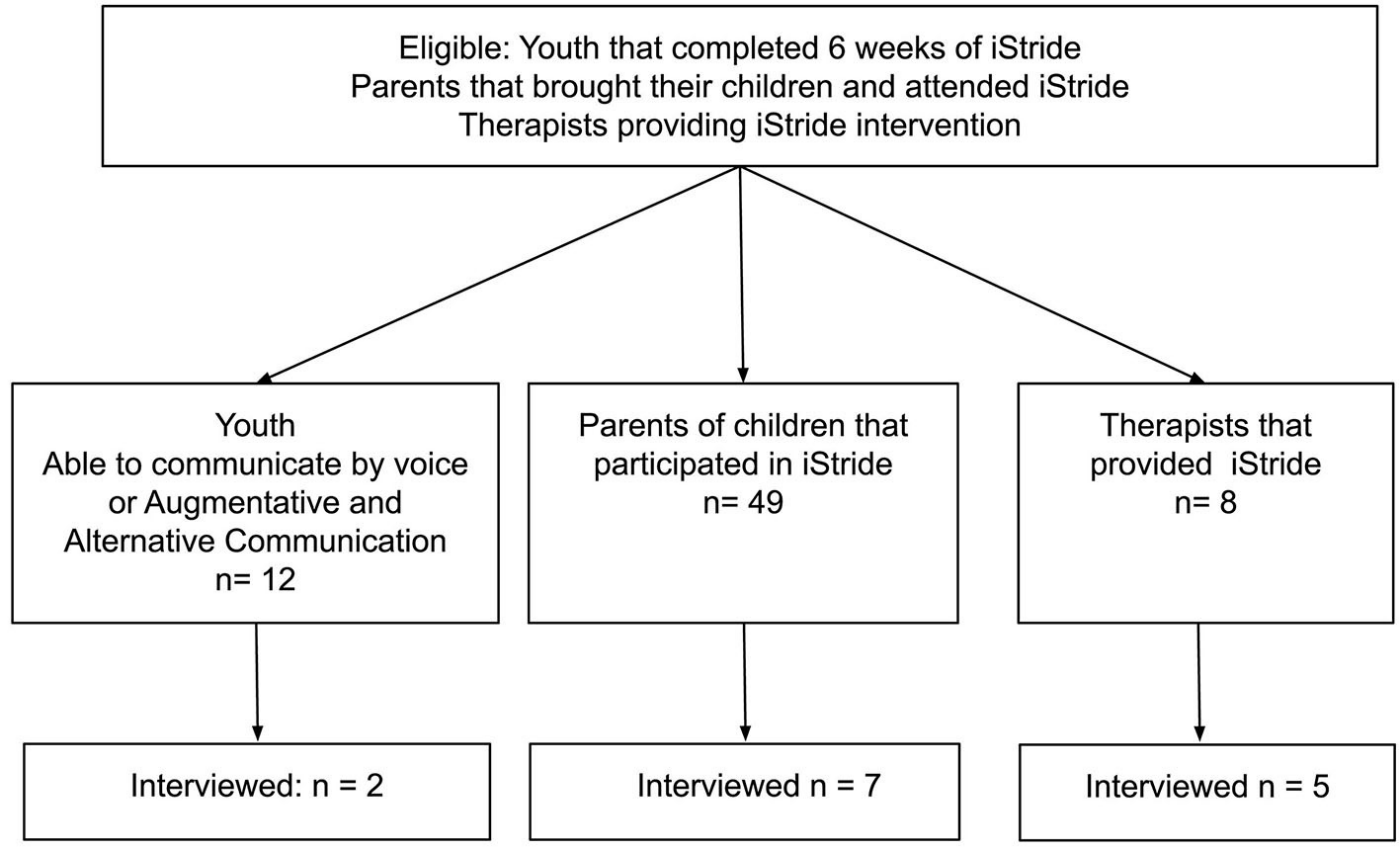
Qualitative study flow chart.

Purposive sampling was used to select participants for this study. This was used to ensure that only children, parents, and therapists involved in the program were sampled. It was considered vital that all invited participants were within 2 weeks of completing the program (Figure 1) (22). This sampling strategy aimed to optimize the trustworthiness of the data so that it did not rely on parents, children, and therapists to recall experiences, rather so that they could reflect on a very recent experience of the intervention. Participants were invited to participate in this study based on their involvement in a 6-week locomotor training intervention (18 h in total). Participants were recruited from the following groups:

Group 1: Parents of children that participated in either the RAGT or PBWSTT group of the locomotor training intervention.
Group 2: Therapists or research assistants who provided the locomotor training intervention throughout the course of the iStride study.
Group 3: Children and youth who participated in the locomotor training intervention. Children were eligible to participate in this study if they (i) were a participant in either the RAGT or PBWSTT group and (ii) had completed the 6-week intervention (18 sessions in total). Children were able to communicate by voice or by augmentative and alternative communication (AAC).

### Data Collection Methods

Semi-structured interviews were carried out over October 2017 and March 2018. Interview guides were specific to each group to reflect appropriate language and jargon containing questions and prompts that were designed to guide the interview in a focused, yet flexible manner. The interview guide was developed and piloted with parents who participated in other mobility interventions in a pediatric rehabilitation outpatient department to obtain feedback of utility prior to use in data collection (Table 1).

**Table 1:** Key topics and prompts in semi-structured interview guides.

**Topic**	**Prompts**		
	**Therapists**	**Parents**	**Children**
Experience	Explain your role in the intervention	Explain the child and parent experience in the intervention	e.g., Tell me about participating in iStride
Body structure and function-related outcomes	Strength, tone, postural control etc.; unintended outcomes.	Strength, tone, postural control etc.; unexpected outcomes.	e.g., Is anything about your body different?
Activity-related outcomes	Mobility, transfers, self-care etc.	Mobility, transfers, self-care etc.	e.g., Can you do new things?
Participation-related outcomes	Attendance and involvement in therapy sessions.	For child and family; Attendance and involvement at home, school, community.	e.g., What is it like when you are at school now?
Contextual factors	Use of robotic devices for therapy; beliefs and attitudes toward intensive intervention; training/skills required	Hospital-based; role of staff; interaction with other families; role demands; intervention equipment	e.g., What was it like using a robot to help you walk?
Impact	Professional practice; recommendations for practice	Goals for child; impact on parent and family; maintaining outcomes.	e.g., How would you explain this to other children?

An exercise physiologist (CW) with expertise in consumer engagement, interviews, and qualitative research in the area of participation in youth with physical disabilities conducted all of the interviews. The interviewer was not involved in any aspect of the intervention provided as part of iStride. The interviews were conducted with parents and children at the conclusion of their 6-week locomotor training intervention (dosage of three, 1 h sessions a week for 6 weeks). The interviews were conducted in the homes of children and parents, or in a private clinic room in the rehabilitation department at a pediatric tertiary hospital (Perth, Australia). Depending on participant preference and availability, interviews with parents also included interviews with youth. Interviews with therapists occurred at the conclusion of the iStride intervention in a private clinic room at the pediatric tertiary hospital. Interviews were approximately 60 min in duration. Audio recordings were used to capture the content of all interviews and field notes were taken.

### Data Analysis

Discussions were transcribed verbatim and compared with field notes taken during interview sessions. Constant comparative coding was used, whereby new codes were compared with those that had emerged from previous interviews through a process of moving back and forth between transcripts. Open coding was applied to each of the interview groups (parents, children, and therapists) whereby three investigators (DP, LW, and AT) generated the initial codes, labeling the meaning units in a spreadsheet on Microsoft Excel. After applying the open coding framework, meaning units were reviewed to merge similar codes and generate themes. Two authors (DP and AT) then completed the thematic analysis over the course of multiple discussion meetings. Following these meetings, the critical friends approach (23), where the data could be challenged and interpreted by an investigator not involved in the generation of meaning units and themes occurred. The coding was combined for the three groups (parents, children, and therapists) with themes grouped into higher-order categories according to discussion areas and reflected broadly the (a) experiences of the intervention and (b) perceived outcomes of the intervention.

### Trustworthiness

Ethical considerations relating to the qualitative component of this trial are largely addressed by having an independent researcher with no previous relationship to trial participants or involvement in the intervention. Several methods to ensure trustworthiness were also undertaken. Credibility was determined by member checking whereby participants reviewed transcripts to confirm the accuracy of their interview (23). In addition, the purposive sampling strategy enabled recent experiences to be explored from participants who were directly involved in the intervention. Comparable conclusions between the three investigators who reviewed the meaning units occurred, providing feedback on and enabling detailed discussions to resolve discrepancies whilst considering any alternative meaning units. Transferability was determined by purposive sampling where research participants were chosen purposively to include children with CP (GMFCS levels III, IV, and V) and their parents and therapists who provided the intervention (22). Dependability was determined by overlap methods where triangulation of data was also undertaken with the quantitative data that was obtained from the randomized controlled trial (24).

## Results

Twelve participants (including seven parents: six females, one male, and five therapists: all female) were interviewed. Combined between the seven parents included six children classified within each of the GMFCS levels III, IV, and V. Pseudonyms are used for each child when they are referred to by therapists, parents, or the child themselves. This includes Adam and Nathan (GMFCS level III), Jackson and Jordan (GMFCS level IV), and Dennis and Stephanie (GMFCS level V). In addition to this, only two of these children were available at the time to be interviewed (Table 2). This included Jackson (GMFCS IV, male, 8 years old, verbal, living at home with both biological parents) and Dennis (GMFCS V, male, 8 years old, using AAC, living at home with both biological parents). The discussions generated 58 pages of 12-point, single spaced text. Themes were grouped into higher-order categories according to discussion areas and reflected broadly the (a) experiences of the intervention and (b) perceived outcomes of the intervention. In the material that follows, we elaborate on these categories, and the supporting tables provide additional meaning unit examples to supplement those presented in the main text.

**Table 2:** Participant characteristics (*n* = 14).

**Characteristic**	**Frequency (%)**
Parents	
Mothers	6 (42.9)
Fathers	1 (7.1)
Children	
GMFCS level IV	1 (7.1)
GMFCS level V	1 (7.1)
Therapists	
Physiotherapist	2 (14.3)
Therapy Assistant	2 (14.3)
Occupational Therapist	1 (7.1)

*GMFCS, Gross Motor Function Classification System*.

### Experiences of the Intervention

Meaning units in this category were grouped according to whether therapists and families were discussing the suitability of the locomotor training (during and beyond the research project), their motivations for participating in locomotor training, and the barriers and facilitators to their participation in the locomotor training. A description of each theme, and sub-themes, are presented in Table 3.

**Table 3:** Feasibility and theme and sub-theme description.

**Theme**	**Theme description**	**Sub-theme**
Acceptability and suitability of locomotor training	The extent to which intensive locomotor training is judged to be suitable to therapists providing the intervention and for participants and their families receiving it, and their perceptions of its utility beyond a research project.	• Intervention length and time • Engagement within sessions • Importance of support • Utility of locomotor training beyond research
Motivations for participating	The extent to which intensive locomotor training is of interest to participants and their families. This includes participant accessibility to activity-based therapy programs that may already be available to them.	• Enjoyment of movement • Increasing activity level
Practicalities and implementation of an intensive therapy program	The personal and environmental barriers and facilitators that affect the implementation and provision of locomotor training and participation in intensive locomotor training.	• Environmental factors • Personal factors
Efficacy/Outcomes	Physical health benefits achieved through intensive locomotor training, including fitness, strength, gross motor, tone and well-being.	• Physical health
	Locomotor training contributes to improved sleep quality and duration.	• Sleep
	Locomotor training induced improvements in mood, confidence, motivation and enjoyment.	• Affect and emotion
	The ability to be more active and independent throughout the day.	• Participation in daily activities

### Suitability of Locomotor Training

Parents and therapists described several structural and content elements of locomotor training intervention. Most notably, they emphasized (a) the intervention length and timing (b), engagement within the sessions, (c) the importance of support, both peer and program, and (d) the utility of locomotor training beyond research.

#### Intervention Length and Timing

Therapists felt that the 6 weeks of intervention offered to families was optimal. One therapist commented,

“A lot of these families are busy, so, so busy so I think 6 weeks is good. Any longer, and I think you probably wouldn't get the attendance.”

Another spoke about the benefits they started to see for the participants toward the end of the 6 weeks,

“Like we quite often don't see change or much change I should say until like week five. We call it magic week five, when all of a sudden it clicks and big things start happening but in saying that by 6 weeks, a lot of the kids are ready for it to be finished, they are tired and they still have school and all their other commitments.”

Parents indicated that for them, a 6-week intervention was achievable, and they could be flexible to meet the needs of their child. However, parents believed that any more than 3 days per week dedicated to an intervention like locomotor training would be too big a commitment. As one parent said,

“Three days a week is nice. I think anything more might be hard to come into the hospital that many times a week. It wouldn't be a problem if the sessions were longer but I think more frequently might be a bit difficult.”

#### Engagement Within the Sessions

Parents also enjoyed how their children engaged with the sessions. When reflecting on the participation of their child during the sessions, Dennis' mother commented,

“Well, they [the sessions] were fun–whether it's [fun] somewhere else I don't know…I think it needs to be fun because it's such hard work so it's good to take the focus off the hard work.” (During this comment, Dennis also nods in agreeance).

Another described the enjoyment of their child in the sessions,

“it was excellent, it was great....yeah just to see the difference in him, and he enjoyed it he really did enjoy it. He had a couple of “I don't want to do this” but more often than not he was quite up for it.”

Children also enjoyed the chance to utilize the technology involved in the locomotor training. Jackson described that the feedback he received from the treadmill as an element of the program that facilitated engagement and enjoyment,

“It [the RAGT device] would give you a little boost wouldn't it because you know that if you were at 80 percent, you needed to work harder to get to 90 percent.”

The suitability of the structure of the interventions, and the positive engagement families had with the intervention was reinforced by the attendance rates that were observed by therapists, as one described,

“…they are obviously getting what they want out of the program and they attended every single session which for some families has been a bit shock to us so it's us adjusting our expectation as well.”

#### The Importance of Support, Both Peer and Program

A common theme across parents and therapists involved in the locomotor training intervention was one of support. Particularly important was both peer support and “top-down” support from the program. Support from the program was a crucial element for the families participating in and adhering to the intervention. One parent commented,

“Sometimes just having that external support and making it more structured, you know you are going to do a bit more.”

Parents also described the benefits they received from spending time with families who had similar experiences to them. One parent commented,

“Being here with other families, it's good for networking with parents, even if it is just for a few minutes.”

This was reinforced by therapists, who noticed the benefits families were getting from being involved in the intervention together. One therapist observed,

“To have a group that is there for 6 weeks interacting with each other...just that social inclusion stuff is really helpful. I feel like it's good for parents to be able to connect with other parents.”

Therapists also described the benefits of peer support for themselves, noting that team dynamics were important to the success of the intervention. Additionally, support from the principal investigator in the form of ongoing education and training was key to ensuring they felt confident and comfortable to deliver the intervention as intended. As one therapist described,

“Just that support I suppose from a senior person like [the principal investigator] or another physiotherapist, to be able to discuss things and programs with them. They will always be there to update things and talk you through it but just having someone there helps.”

#### The Utility of Locomotor Training Beyond Research

Parents and therapists were all able to see the utility of locomotor training beyond the scope of the research project they were involved in. One parent commented,

“If another block is offered again maybe not even the 6 weeks maybe 2 or 3 weeks or something, I'm sure there would be more parents like me who just want those things happening in the future.”

Therapists described the positive benefits they perceived for the families involved in locomotor training. One therapist observed,

“Parents were so excited I think just seeing their children walk. I never understood what it might be like and now that I have a child and watching her walk, walking was such a milestone. For these parents with children with disability who maybe miss so many milestones compared to siblings and peers to see them walking you would see such joy on their faces, both the children and the parents.”

When asked about the continuity of locomotor training beyond the research project, one therapist commented,

“Yes 100 percent, even just going alone from what parents say and want, let alone what we can see happening… All people want is that maintenance and that its ongoing in the future and they are desperate for it.”

### Motivations for Participating in Locomotor Training

In this theme, parents, predominantly, along with children, described the motivations for their participation in locomotor training. Meaning units spanned (a) the enjoyment of the child of movement-based activities and (b) wanting to increase the activity levels of their children.

#### Enjoyment of Movement

Parents described that despite having movement limitations, their children loved having the opportunity to move their bodies. One parent commented,

“Jordan does love movement, which is a big thing for him. It is probably one of his biggest drivers - is movement. So obviously he can't walk, he has a standing frame and a walking frame and so a walking clinic was just ideal for him.”

Parents also described increasing the activity level of their child as a primary motivation for participating in locomotor training, in order to maintain or improve their mobility and/or fitness. On the issue of maintaining or improving mobility, one parent commented,

“We just want to keep Nathan mobile, get his confidence [up] and build up his endurance and that is our goal...If there are things there to help us do that, we will always try to do those things.”

#### Increasing Activity Level

The motivation to improve or maintain fitness levels was important for parents, as they perceived that this was of benefit to the overall health and well-being of their child. As one parent described,

“One of the goals here was fitness you know it wasn't therapy. My goal was fitness because I knew it would get her fitter and I knew that was declining and if anything is going to get it will be pneumonia or some nasty bug. So I never saw it as free therapy I saw it as a chance for exercise, a chance for Stephanie to develop her lungs and get a bit fitter.”

Jackson cited improved fitness as a motivation for participating in locomotor training. A notable comment by Jackson was,

“Because when my body is tired I have to carry him in my wheelchair and he is very heavy.”

### Barriers and Facilitators to Participation in Locomotor Training

Parents and therapists described the (a) environmental and (b) personal factors that affected the implementation and provision of locomotor training.

#### Environmental Factors

For parents, organizing the attendance of their child at locomotor training was sometimes considered a logistical challenge;

“I don't know about other mums, but it's stressful.”

However, parents also reflected that the provision of parking by the study team, for families involved in the study sessions, helped ease aspects of the logistical burden. One parent commented,

“I mean it's [hospital where the study took place] far, but you know…having a parking space was really good. Yes that was a godsend. It takes a lot of the stress out of it....you can just get in the car and leave and you don't have to allow and extra 15 min to find parking.”

Parents also commented that as their children were most attentive during schooling hours, they would often miss parts of the school day to attend locomotor training. Whilst this was considered a challenge for some families, in most instances, there was a mutual understanding between school and family that locomotor training would be of benefit to the child,

“So yeah we did miss [school] but I mean the school is quite good about it they understand that it [locomotor training] is important to us.”

#### Personal Factors

From the perspectives of both parents and therapists, relationships between program staff and families were seen as positive influencers on locomotor training adherence. Consistency in service delivery helped to build rapport, as described by one therapist,

“There isn't stopping and starting, we get to know the clients and how to read them as to when they need a break and what works best for them.”

Parents also commented that therapists made them feel comfortable and confident that the needs of their child were met,

“They were just really encouraging and they were all really understanding and they just know their stuff…I felt she was safe in their hands which is a big deal with a childlike Stephanie.”

Relationships with program staff were highly influential on engagement during sessions and enjoyment of the locomotor training. As one parent commented,

“They made it fun for him so they had his music and they would always give him goals like let's get to 100m then 200m. It suits Jackson's personality that if you give him something, he's got to get there.”

Several therapists described the physicality of delivering the locomotor training intervention as a challenge, particularly with regards to the manual handling and positioning of participants. One therapist commented,

“Yeah it is very energy intensive and physical. We are quite good at managing that, especially with the treadmill. That is the real physical part, we are very good at rotating to work out both sides of your arm and back and give your wrists a break when you are supporting from behind and [PI] is really good at splitting up who is doing too much on one day. I think at the moment we are sometimes doing four a day and that is probably the limit in terms of stress on your body and being bent over in that position the whole time.”

However, therapists also explained that the physicality of the intervention ultimately, in their opinions, facilitated positive outcomes for participants. As one therapist reflected,

“All their walking seems to be a lot easier, they are stronger and our jobs are a lot easier as they progress because they have gotten stronger and they can initiate so much more themselves.”

### Perceived Outcomes of the Intervention

Four themes were categorized as an outcome of participation in locomotor training. Specifically, as a result of participation in the locomotor training intervention, parents and therapists reported participant outcomes related to (a) physical health, (b) sleep, (c) affect and emotion, and (d) ambulation in daily activities. A description of each theme, and additional meaning units, are presented in the Supplementary Material.

#### Physical Health

Within this theme, parents and therapists highlighted improvements in strength, gross motor function, and overall physical well-being because of the involvement of participants in locomotor training. At the conclusion of the intervention, most of the parents described improvements in the strength of their child. As one parent commented,

“He got the strength, you know more strength, and he got the confidence, which I think is what he is lacking.”

The benefits seen from locomotor training also extended to gross motor activities such as walking. As one parent described,

“My primary reason for participating was he was going to walk longer, he was going to walk better he was going to be stronger. And all three of those things happened.”

Therapists also described further improvements in other gross motor activities, one observed

“I think the big thing was that for the GMFCS V kids it was having an impact on their rolling and functional mobility which was interesting.”

Another commented,

“And just sitting, their ability to sit upright has improved with a lot of them who initially couldn't sit unsupported and by the end of it some of them have been able to which is really cool.”

Parents and therapists noticed that, for participants who were more reliant on wheelchairs throughout the day, the impact of locomotor training was different, relating more acute changes in tone and movement patterns. One parent said,

“So she started the day with a lot of uncontrolled movements but after her [locomotor training] session her uncontrolled movements reduced significantly. So that's on a hard day, it made it better.”

The notion of acute changes in tone were also noted by therapists, as one described,

“We see changes in tone as well because obviously we are very hands on especially on the treadmill when we are facilitating their stepping you can definitely feel over time the tone changing.”

Pre-existing respiratory and digestion issues, commonplace among this population, seemed to be positively impacted by locomotor training. One parent described,

“My thoughts were previous to the study [she] was getting sick every 2–3 weeks and they [were] always an upper respiratory sickness, she often has antibiotics to get over a sickness and fever. Her breathing at night was becoming more problematic with audible strider-like breathe and mum and dad have to sit up until she goes to bed with her…So all that got better. There has been no sickness so far and no antibiotic use. Previous to this Stephanie has been sick every 3 weeks and has missed school. Her overall temperature seems stable- previous to this it wasn't. She has had no [paracetamol] over this period...it's the walking”.

#### Sleep

Parents reported that participation in the locomotor training contributed to improved sleep quality and duration for their children and, in some instances, themselves. For example, one parent commented,

“Sleeping through is great for everybody.....So I don't have to get up and I don't have broken sleep, she doesn't have broken sleep it's heaps better.”

Parents also reported that the easing of respiratory symptoms also contributed to improved sleep for their children. As one parent described,

“Better breathing especially at night, constant sleeping through the night with no waking in the middle of the night. No sickness which is hugely significant, and better ability to cough.”

Therapist observations mirrored these changes, with regards to the easing of respiratory symptoms and the impact on sleep. For instance, one therapist said,

“They've been sleeping better, easier to change, the girl that became easier to change didn't really sleep through the night but then after the training was able to sleep through the night... and Mum was over the moon.”

#### Affect and Emotion

Parents and therapists described participants having improvements in mood, confidence, and motivation following their involvement in locomotor training. Regarding mood, parents perceived their children to be generally happier and calmer following their involvement in locomotor training. As one parent said,

“…he's waking up happy and excited for what the day is going to bring and I think he is a little bit hopeful now that every appointment is going to be a [locomotor training] appointment.”

Improvements in mood also extended to parents, as they were able to see the benefits the locomotor training had for their family. As one parent explained,

“Oh it's made us happy, well we have always celebrated Adam's little achievements we just seem to celebrate them a little more...And the fact that he is faster…. Tensions are down a little more too. You know, first thing in the morning when we're trying to get somewhere, it's just a bit easier.”

Participation in locomotor training led to participants feeling more confident in themselves and their abilities, as described by parents and therapists. One therapist commented,

“A lot [of participants] start out with “I can't do this” or “this is hard I don't want to do this. “By the end of the program it's “I can do this it's amazing look what I did this week” and “I did this at school today.” One boy who does cross country, he was like “I did 1.2 km in 9 min today”... Even then he would fall, but he would get back up himself. Mum was in tears and the teachers were in tears.... their perceptions of themselves and what they are able to do is really cool.”

This confidence, in turn, seemed to contribute to improvements in the motivation of participants to be involved in activities both within and outside of school. As one parent described,

“he was faster and stronger and he wanted to get in his walker more so that was cool” and another, “We've seen better performance at school, more alert, and better attention.”

This notion was reinforced by therapists, as one therapist commented,

“What we have heard from parents and kids is that they've been more involved in sports. One of the girls was not involved in any kind of community activity outside of school and now she comes in and she is like “I'm going to sign up for wheelchair basketball and I'm going horse-riding” which is awesome and that's the whole point isn't it.”

#### Ambulation in Daily Activities

In this theme, parents and therapists highlighted the impact that locomotor training had on the ability of participants to be more active and independent throughout the day. Endurance and speed of walking improved the ability of participants to ambulate in the community and at school. As one parent described,

“Of course, the biggest thing from school is the distance between the bus stop and nappy changing area and his classroom is huge so before they would have to set aside 20 min just to get his nappy changed and back again. Whereas now they probably get it all done in about 8 min because he's walking and thinking “I'm doing it.” The teacher is happy because she actually gets to spend more time with him. That transition from the bus stop to the classroom, every day he does it he gets faster”.

Parents also reported an impact on activities within the home, as one parent said,

“what I would do is I would get Jordan to walk at home you know little journeys from his bedroom to the lounge room I would get him to walk down the hall and prior to [the locomotor training program] he would occasionally put down one foot and then lifting both feet and basically I would carry him and he would put down one foot but now he is doing two steps most of the time again.”

## Discussion

This qualitative study describes the experience of intensive locomotor training from the perspective of therapists and parents of children with CP. It was important to describe these experiences because intensive or more highly dosed interventions do require more time commitment from children and their families. By understanding both the experience of and perceived outcomes of locomotor training, the implementation of locomotor training beyond the research context can be shaped to facilitate the translation of research into the “real-world” in children with CP functioning within GMFCS levels III, IV, and V.

The optimum treatment dosage for locomotor training for children with CP is yet to be established. The current literature has reported positive mobility outcomes with treatment dosages that range from 2 to 5 days a week over a period of 2 to 25 weeks (25, 26). The wide range of treatment dosages reported in the literature is challenging for both therapists and parents who need to plan for a more highly dosed intervention. Long treatment durations may potentially be effective but can be expensive, place undue stress on families, and impacts schooling. Therefore, an important question is raised: what is the minimum duration that will facilitate meaningful changes? In this study, therapists described observable mobility changes from week 5. For parents, they reported that they felt ready to finish the program by week 6. Given that the current physical activity dosage recommends a dosage of three times a week for 8 weeks (27), it is likely that short intervention durations will result in minimal meaningful physical changes. Yet, when considering the reported impact of an intensive intervention on families, longer intervention durations (beyond 6 weeks) may be burdensome for children and their parents (28). Although no specific recommendations can be made from this qualitative study about optimum treatment dosage, the reported experiences from this study do highlight the importance of having conversations with families about the balance between optimal treatment dosages, meaningful physical outcomes, and the impact of lengthy appointment schedules on the family unit and education. This qualitative study provides a useful starting point to initiate discussions on balancing all of these factors. An important future research direction would be to determine optimal treatment dosages and to evaluate the cost-effectiveness of more highly dosed interventions in relation to outcomes.

Parents and therapists reported outcomes important to their family and child beyond what is traditionally measured in motor-based interventions. In children and youth with CP, controlled trials have largely focused on activity capacity motor outputs, such as improvements in walking speed (9, 25) and endurance (29). However, reports in this study are consistent with previously reported uncontrolled trials and case series which provided early evidence of outcomes beyond walking speed and endurance which include improved confidence and mood (7), improved weight acceptance and transfers (30), reduced caregiver support, improved skills in gait trainers, and improved bowel function (31). In addition to this, parents provided the context of the perceived improvements that were meaningful to them. For example, improvements in walking endurance were contextualized within the school environment, where improved walking speed and endurance enabled more time in class. Within the home, improvements in walking meant that morning routines were faster, with children being “happier” and “calmer,” having a positive impact on family dynamics. Collaborative goal setting is highlighted here, and these parent and therapist experiences provides a much-needed prompt to consider goals that are beyond motor outputs alone.

The value of engaging in physical activity, particularly for children that tend to spend more time sedentary, is reinforced by parents and therapists as they reported outcomes that directly reflect the known effects of physical activity (32, 33). This included improvements in respiratory function which was reported to impact their sleep quantity and quality both for themselves and their child. Improved confidence was also reported, and parents related this to a more positive outlook and greater motivation to try new things to engage in community activities. Furthermore, parents noted that the increased activity levels during the locomotor training period were linked to improvements in mood. The link between activity and mental health is well established with low levels of physical activity being associated with poorer psychological well-being (34), and higher levels of physical activity being associated with improvements in the self-esteem and psychological health of children (34–37). For children with CP classified within GMFCS levels III, IV, and V, locomotor training provides an opportunity to improve and preserve both physical and mental health.

Based on the outcomes of this qualitative study, we recommend that future physical activity or motor-based studies consider the inclusion of children with greater equipment needs (GMFCS levels III, IV, and V) along with the inclusion of outcome measures on sleep quality and quantity, quality of life, respiratory outcomes, participation measures, mental health outcomes, and caregiver support. The breadth of outcomes reported by parents and therapists consolidates the importance of taking a 24-h “whole day matters” approach where the distribution of physical activity, sedentary behavior, and sleep impacts the health and well-being of a child (38). Further to this, quantifying the impact of treatments requires tailoring and the adoption of individualized measures is necessary to objectively and reliably determine meaningful activity and participation outcomes. Measures such as the Canadian Occupational Performance Measure and the Goal Attainment Scale are particularly relevant and are likely to reflect specific and meaningful changes for the child and their family within a “whole day matters” approach (39).

There are some practical considerations that can be considered for future research and intervention provision. Parents and therapists both agreed accessibility (such as parking accessibility and scheduled session times) to the intervention are important considerations. Enjoyment or fun should be considered for both children during the sessions and for parents who may benefit from the company of other parents of children with CP. Parent-to-parent peer support provides opportunities for parents to share and explore their feelings and experiences with others who have similar journeys (40). Finally, for therapists providing the intervention, support and training are integral to optimize treatment fidelity and ensure co-ordinated care for children with higher physical needs.

The current study has several strengths which include a qualitative design with robust methods to enhance trustworthiness. The interview guide was developed with family engagement through a pilot study that was conducted prior to this qualitative study. The inclusion of therapists who were involved in providing the intervention, along with one child classified within GMFCS IV who was able to participate verbally, provided a different perspective. This research also provides insights from those involved in the provision of high dosed interventions for children with high physical assistance and equipment needs.

The main limitation is that only two children were interviewed, one of which was a child that used AAC and tended to nod in agreeance when his mother spoke about her experiences rather than answer questions directly about his own experiences. Gaining more perspectives of children and youth on their experiences, particularly for a more highly dosed intervention, would be an important future direction because it has the potential to influence knowledge translation of interventions in the community. Future studies should also consider designing specific questions for children using AAC and, where possible, conducting the interview without their parents in the room. However, we acknowledge that this may be challenging for children particularly if they are classified within GMFCS levels IV and V. Another limitation is that the timing of the interview was captured following the intervention which means any outcomes of retention were not obtained. Generalisability is therefore impacted as a result as is the fact that this study was only limited to one site. Finally, another limitation is that the effect of the intervention on schooling was not specifically explored. Given both the intensity of such a program and the uncertainty around recommended intervention dosages, exploring the impact of such a program on educational experiences is an important future direction.

## Conclusion

From the perspectives of parents and therapists, locomotor training provided with a high dosage over 6 weeks provided children with CP classified as functioning at GMFCS levels III, IV, and V an opportunity to engage in physical activity. Engaging in physical activity was a consistent motivator, and improvements in physical health, sleep, affect, and emotion and ambulation in daily activities were meaningful outcomes for parents. Future studies are needed to determine the optimal dosage required for optimal outcomes and the inclusion of a broader range of health and well-being outcomes alongside individualized measures on activity and participation are needed to better understand and quantify meaningful changes for the child beyond motor-based outcomes.

## Data Availability Statement

The original contributions presented in the study are included in the article/Supplementary Material, further inquiries can be directed to the corresponding author.

## Ethics Statement

The studies involving human participants were reviewed and approved by Curtin University Human Ethics Research Committee. Written informed consent to participate in this study was provided by the participants' legal guardian/next of kin.

## Author Contributions

DP and CE planned the iStride trial, wrote the protocol, obtained funding, and oversaw the study. CW conducted the interviews with all participants. DP and AT carried out the initial analysis of the manuscript with CW being the critical friend after the initial analysis. DP and AT drafted the manuscript and revised it in response to revisions from all authors. All authors have read and approved the final manuscript.

## Funding

This research was supported by the Telethon7 Trust and the Perth Children's Hospital Research Fund 2014 (Round 3).

## Conflict of Interest

The authors declare that the research was conducted in the absence of any commercial or financial relationships that could be construed as a potential conflict of interest.

## Publisher's Note

All claims expressed in this article are solely those of the authors and do not necessarily represent those of their affiliated organizations, or those of the publisher, the editors and the reviewers. Any product that may be evaluated in this article, or claim that may be made by its manufacturer, is not guaranteed or endorsed by the publisher.
