# Supplementary material for: The Experience of Locomotor Training From the Perspectives of Therapists and Parents of Children With Cerebral Palsy
Source: Front Rehabil Sci. 2021 Dec 2;2:740426. doi: 10.3389/fresc.2021.740426 (PMC9397752; doi:10.3389/fresc.2021.740426)
Supplement: Supplementary file 1 [file Table_1.docx]

| Theme | Sub-theme | Exemplar Meaning Units |
| --- | --- | --- |
| **Suitability of locomotor training** | Intervention length and timing | “A lot of these families are busy, so, so busy so I think six weeks is good. Any longer, and I think you probably wouldn’t get the attendance.” (T) |
|  |  | “Like we quite often don’t see change or much change I should say until like week five. We call it magic week five, when all of a sudden it clicks and big things start happening but in saying that by six weeks, a lot of the kids are ready for it to be finished they are tired and they still have school and all their other commitments.” (T) |
|  |  | “on the family it wasn’t too bad because it was all during work hours so it was just me and him. I told [them] as long as it fitted…… I had to be home to be here for [my other son].....anytime it’s available I’ll bring him to you as long it’s between 8-3.” (P) |
|  |  | “Three days a week is nice. I think anything more might be hard to come in to the hospital that many times a week. It wouldn’t be a problem if the sessions were longer but I think more frequently might be a bit difficult.” (P) |
|  | Engagement within sessions | “Well, they [the sessions] were fun - whether it’s [fun] somewhere else I don’t know…I think it needs to be fun because it’s such hard work so it’s good to take the focus off the hard work.” (P) |
|  |  | “it was excellent, it was great....yeah just to see the difference in him, and he enjoyed it he really did enjoy it. He had a couple of “I don’t want to do this” but more often than not he was quite up for it.” (P) |
|  |  | “It [the RAGT device] would give you a little boost wouldn’t it because you know that if you were at 80 percent, you needed to work harder to get to 90 percent.” (Y) |
|  |  | “Some of them have really surprised us in that we didn’t think they would attend due to past history of not attending appointments and you kind of go in with those expectations but they are obviously getting what they want out of the program and they attended every single session which for some families has been a bit shock to us so it’s us adjusting our expectation as well.” (P) |
|  | Importance of support (peer and program) | “Being here with other families, it’s good for networking with parents, even if it is just for a few minutes.” (P) |
|  |  | “To have a group that is there for 6 weeks interacting with each other...just that social inclusion stuff is really helpful. I feel like it’s good for parents to be able to connect with other parents.” (T) |
|  |  | “The team has been structured well, the communication between the team is good and works well.” (T) |
|  |  | “It [this project] has made me very, very passionate about what we want to achieve from it and it’s so big there are so many different avenues you could go down. It’s not just about locomotor stuff anymore it’s not just about walking or standing it’s about so many other things.” (T) |
|  |  | “Sometimes just having that external support and making it more structured, you know you are going to do a bit more.” (P) |
|  |  | “I think the burden of care eases in some respects because they [parents] can see them [their child] progressing and what their potential is... Some of them have such full on jobs and days with the kids it’s nice for them to be able to step back and just to observe some of it but also be able to go and have a bit of chill time and came back and know that they are in good hands.” (T) |
|  |  | “Just that support I suppose from a senior person like [the principle investigator] or another physiotherapist, to be able to discuss things and programs with them. They will always be there to update things and talk you through it but just having someone there helps.” (T) |
|  | Utility of locomotor training beyond research | “If another block is offered again maybe not even the 6 weeks maybe 2 or 3 weeks or something, I’m sure there would be more parents like me who just want those things happening in the future.” |
|  |  | “All their walking seems to be a lot easier, they are stronger and our jobs are a lot easier as they progress because they have gotten stronger and they can initiate so much more themselves.” |
|  |  | “Parents were so excited I think just seeing their children walk. I never understood what it might be like and now that I have a child and watching her walk, walking was such a milestone. For these parents with children with disability who maybe miss so many milestones compared to siblings and peers to see them walking you would see such joy on their faces, both the children and the parents.” |
|  |  | “Yes 100 percent, even just going alone from what parents say and want, let alone what we can see happening. Providing we had all the equipment and the team I don’t see any reason why it wouldn’t work. All people want is that maintenance and that it’s ongoing in the future and they are desperate for it.” |
| Motivations for participating in locomotor training | Child’s enjoyment of movement-based activities | “[child’s name] does love movement, which is a big thing for him. It is probably one of his biggest drivers is movement. So obviously he can’t walk, he has a standing frame and a walking frame and so a walking clinic was just ideal for him.” (P) |
|  |  | “We just want to keep him mobile, get his confidence [up] and build up his endurance and that is our goal...If there are things there to help us do that, we will always try to do those things.” (P) |
|  | Increase activity levels | “One of the goals here was fitness you know it wasn’t therapy. My goal was fitness because I knew it would get her fitter and I knew that was declining and if anything is going to get it will be pneumonia or some nasty bug. So I never saw it as free therapy I saw it as a chance for exercise, a chance for her to develop her lungs and get a bit fitter.” (P) |
|  |  | “Because when my body is tired I have to carry him in my wheelchair and he is very heavy.” (Y) |
| Barriers and facilitators to participation in locomotor training | Environmental | “I don’t know about other mums, but it’s stressful. So if there was maybe northern and something maybe southern maybe but I don’t know if that could work. Something like that would be good. Because parking is a nightmare I mean even though [she] had one someone parked in there once and I couldn’t get it so we were a bit late one time. And you’ve got a wheelchair and a walker.” (P) |
|  |  | “I mean it’s [hospital where the study took place] far, but you know…having a parking space was really good. Yes that was a godsend. It takes a lot of the stress out of it....you can just get in the car and leave and you don’t have to allow and extra 15 minutes to find parking.” (P) |
|  |  | So yeah we did miss [school] but I mean the school is quite good about it they understand that it [locomotor training] is important to us.” (P) |
|  |  | “I’m definitely open to the social side of it too. I think my son feeds off that and it motivates him and he likes noise and all the commotion so yeah definitely [the group].” (P) |
|  |  | “[in a group] It would have been distracting if other people were on other things cos then I would just look around and see what other people were doing.” (Y) |
|  |  | “Most of the kids were pretty good. I would say the older ones were a bit better. Some of the younger children we saw or some kids who had intellectual impairment were easily distractible and they found it harder to concentrate particularly with treadmill training. So to keep them on track was quite challenging and took a bit of work.” (T) |
|  |  | “There isn’t stopping and starting, we get to know the clients and how to read them as to when they need a break and what works best for them.” (T) |
|  |  | “They were just really encouraging and they were all really understanding and they just know their stuff. I used to take [her] iPad in just so we can use it as a bit of a motivator and during the transfers she is looking at something and she is distracted. They got on to that pretty well, I felt safe if I had to leave. I felt she was safe in their hands which is a big deal with a child like [name].” (P) |
|  |  | “ We just need to be quite strict and have game plan and set real clear rules otherwise they do try and avoid and get out of sessions but the majority stick with it and we just have to change little things to make it newer and fresher as they go along.” (T) |
|  |  | “They made it fun for him so they had his music and they would always give him goals like let’s get to 100m then 200m. It suits [child’s name] personality that if you give him something, he’s got to get there.” (P) |
|  |  | We’re a good team that works well together. We all know our roles and we all talk to each other prior to the kids coming in you know “who is going to what” “who is going to step out when they aren’t needed” there is no fluffing around kind of thing so it’s smooth and I think parents appreciate that.” (T) |
|  |  | “Yeah it is very energy intensive and physical. We are quite good at managing that, especially with the treadmill. That is the real physical part, we are very good at rotating to work out both sides of your arm and back and give your wrists a break when you are supporting from behind and [PI] is really good at splitting up who is doing too much on one day. I think at the moment we are sometimes doing four a day and that is probably the limit in terms of stress on your body and being bent over in that position the whole time.” (T) |
|  |  | “All their walking seems to be a lot easier, they are stronger and our jobs are a lot easier as they progress because they have gotten stronger and they can initiate so much more themselves.” (T) |
|  |  | “having the fitness component is good and having the heart rate monitor gives us the confidence to push them and their fitness.” (T) |
|  |  | “I think what it shows us even more is that especially with the GMFCS V their heart rate would skyrocket on the treadmill and it just made you appreciate how much energy they are expending and that we needed to be more mindful of that.”(T) |
| Perceived outcomes of the intervention | Physical Health | “He got the strength, you know more strength, and he got the confidence, which I think is what he is lacking.” (P) |
|  |  | “My primary reason for participating was he was going to walk longer, he was going to walk better he was going to be stronger. And all three of those things happened.”(P) |
|  |  | “I think the big thing was that for the GMFCS 5 kids it was having an impact on their rolling and functional mobility which was interesting.”(T) |
|  |  | “And just sitting, their ability to sit upright has improved with a lot of them who initially couldn’t sit unsupported and by the end of it some of them have been able to which is really cool.” (T) |
|  |  | “My thoughts were previous to the study [she] was getting sick every 2-3 weeks and they [were] always an upper respiratory sickness, she often has antibiotics to get over a sickness and fever. Her breathing at night was becoming more problematic with audible strider-like breathe and mum and dad have to sit up until she goes to bed with her…So all that got better. There has been no sickness so far and no antibiotic use. Previous to this [she] has been sick every 3 weeks and has missed school. Her overall temperature seems stable- previous to this it wasn’t. She has had no [paracetamol] over this period ...it's the walking” (P) |
|  | Sleep | “Sleeping through is great for everybody.....So I don’t have to get up and I don’t have broken sleep, she doesn’t have broken sleep it’s heaps better.” (P) |
|  |  | “Better breathing especially at night constant sleeping through the night with no waking in the middle of the night. No sickness which is hugely significant, and better ability to cough.” (P) |
|  |  | “They’ve been sleeping better, easier to change, the girl that became easier to change didn’t really sleep through the night but then after the training was able to sleep through the night... and Mum was over the moon.”(T) |
|  | Affect and emotion | “…he’s waking up happy and excited for what the day is going to bring and I think he is a little bit hopeful now that every appointment is going to be a [locomotor training] appointment.” (P) |
|  |  | “Oh it’s made us happy, well we have always celebrated [child’s name]’s little achievements we just seem to celebrate them a little more...And the fact that he is faster…. Tensions are down a little more too. You know, first thing in the morning when we’re trying to get somewhere, it’s just a bit easier.” (P) |
|  |  | “A lot [of participants] start out with “I can’t do this” or “this is hard I don’t want to do this. “By the end of the program it’s “I can do this it’s amazing look what I did this week” and “I did this at school today. “ One boy who does cross country, he was like “I did 1.2km in 9min today”... Even then he would fall, but he would get back up himself. Mum was in tears and the teachers were in tears.... their perceptions of themselves and what they are able to do is really cool.” (T) |
|  |  | “he was faster and stronger and he wanted to get in his walker more so that was cool” and another, “We’ve seen better performance at school, more alert, and better attention.” (P) |
|  |  | “What we have heard from parents and kids is that they’ve been more involved in sports. One of the girls was not involved in any kind of community activity outside of school and now she comes in and she is like “I’m going to sign up for wheelchair basketball and I’m going horse-riding” which is awesome and that’s the whole point isn’t it.” (T) |
|  | Ambulation in daily activities | “Of course, the biggest thing from school is the distance between the bus stop and nappy changing area and his classroom is huge so before they would have to set aside 20 minutes just to get his nappy changed and back again. Whereas now they probably get it all done in about 8 minutes because he’s walking and thinking “I’m doing it. “ The teacher is happy because she actually gets to spend more time with him. That transition from the bus stop to the classroom, every day he does it he gets faster”. (P) |
|  |  | “what I would do is I would get him to walk at home you know little journeys from his bedroom to the lounge room I would get him to walk down the hall and prior to [the locomotor training program] he would occasionally put down one foot and then lifting both feet and basically I would carry him and he would put down one foot but now he is doing two steps most of the time again.” (P) |
|  |  | “So she started the day with a lot of uncontrolled movements but after her [locomotor training] session her uncontrolled movements reduced significantly. So that’s on a hard day, it made it better.” (P) |
|  |  | “We see changes in tone as well because obviously we are very hands on especially on the treadmill when we are facilitating their stepping you can definitely feel over time the tone changing.” (T) |

(T): Therapists; (P): Parents; (Y): Youth
